# RhizoVision Crown: An Integrated Hardware and Software Platform for Root Crown Phenotyping

**DOI:** 10.34133/2020/3074916

**Published:** 2020-02-15

**Authors:** Anand Seethepalli, Haichao Guo, Xiuwei Liu, Marcus Griffiths, Hussien Almtarfi, Zenglu Li, Shuyu Liu, Alina Zare, Felix B. Fritschi, Elison B. Blancaflor, Xue-Feng Ma, Larry M. York

**Affiliations:** ^1^Noble Research Institute, LLC, 2510 Sam Noble Parkway, Ardmore, OK 73401, USA; ^2^Division of Plant Sciences, University of Missouri, Columbia, MO 65201, USA; ^3^Crop and Soil Sciences, University of Georgia, Athens, GA 30602, USA; ^4^Texas A&M AgriLife Research, Texas A&M University System, Amarillo, TX 79106, USA; ^5^Department of Electrical and Computer Engineering, University of Florida, Gainesville, FL 32601, USA

## Abstract

Root crown phenotyping measures the top portion of crop root systems and can be used for marker-assisted breeding, genetic mapping, and understanding how roots influence soil resource acquisition. Several imaging protocols and image analysis programs exist, but they are not optimized for high-throughput, repeatable, and robust root crown phenotyping. The RhizoVision Crown platform integrates an imaging unit, image capture software, and image analysis software that are optimized for reliable extraction of measurements from large numbers of root crowns. The hardware platform utilizes a backlight and a monochrome machine vision camera to capture root crown silhouettes. The RhizoVision Imager and RhizoVision Analyzer are free, open-source software that streamline image capture and image analysis with intuitive graphical user interfaces. The RhizoVision Analyzer was physically validated using copper wire, and features were extensively validated using 10,464 ground-truth simulated images of dicot and monocot root systems. This platform was then used to phenotype soybean and wheat root crowns. A total of 2,799 soybean (*Glycine max*) root crowns of 187 lines and 1,753 wheat (*Triticum aestivum*) root crowns of 186 lines were phenotyped. Principal component analysis indicated similar correlations among features in both species. The maximum heritability was 0.74 in soybean and 0.22 in wheat, indicating that differences in species and populations need to be considered. The integrated RhizoVision Crown platform facilitates high-throughput phenotyping of crop root crowns and sets a standard by which open plant phenotyping platforms can be benchmarked.

## 1. Introduction

Roots serve as the interface between the plant and the complex soil environment with key functions of water and nutrient extraction from soils [1, 2]. Root system architecture (RSA) refers to the shape and spatial arrangement of root systems within the soil, which plays an important role in plant fitness, crop performance, and agricultural productivity [1, 3, 4]. RSA is shaped by the interactions between genetic and environmental components and influences the total volume of soil that roots can explore [3]. Many root phenes (or elemental units of phenotype [4–6]) shape the final RSA, including the number, length, growth angle, elongation rate, diameter, and branching of axial and lateral roots [7]. Understanding the contribution of RSA phenes to crop performance is of key importance in food security and for breeding of more productive and resilient varieties in a changing environment.

Because roots are hidden underground and require considerable effort to characterize, research on plant roots lags behind that of the aboveground organs [8], and the genetic and functional basis of RSA remains obscured [9]. Phenotyping is a major bottleneck in research, and a lack of efficient methods for collecting root phenotypic data is limiting progress in using RSA for genetic studies and breeding for root ideotypes [10, 11]. In recent years, there has been a shift to image-based phenotyping for enabling relatively high throughput and accurate measurements of roots. Many of the platforms use 2D imaging with cameras and involve the use of seedlings on agar plates, germination paper, or fabric cloth in bins [11]. Despite the usefulness of controlling environmental parameters for characterization of root phenotypes, studies of roots of field-grown plants better represent the agricultural systems in which they ultimately grow.

Weaver and colleagues [12, 13] pioneered methods for excavating, drawing, and photographing root systems that have been widely used for more than half a century [14]. These classical methods were since modified [15] with the use of water to remove soil particles from the root systems on a large scale and the use of high pressure air to penetrate soil pores while leaving roots intact [16]. Hydropneumatic root elutriation was developed by Smucker et al. [17] to provide a rapid and reproducible approach for separating roots from soil of field-collected soil core samples with minimal damage. Traditional excavation methods are most suited for trees and shrubs as the root system of wooden species is generally stronger and more resistant to breaking than the finer roots of grasses or annual crops [14]. Other field root phenotyping methods include minirhizotrons and soil coring, which both require a large amount of physical labor and set-up time [14, 18, 19]. More recently, nondestructive root phenotyping methods such as ground penetrating radar and electrical resistance tomography have shown promise; however, both techniques only provide indirect assessments of root length, do not provide RSA features, and have not been shown to be ready for reliable, large-scale use [20, 21].

Over the past 10 years, root crown phenotyping [22] has emerged as one of the more common field-based root phenotyping methods and is characterized by excavation of the top portion of the root system, removal of soil, and measurements, by a variety of means. The definition of root crown as the top portion of the root system in this research is extended from the earlier use of this terminology which refers to the site where the root system transitions to the shoot [23]. The term “root crown” should not be confused with “crown root,” which refers to belowground nodal roots. Root crown phenes, such as nodal root number [4, 24, 25] and growth angle [25–28], have been widely reported to correlate with crop aboveground biomass, nutrient content, or grain yield. The work of Grift et al. [29] may be the earliest published example of root crown phenotyping in a high-throughput capacity. Root crown phenotyping was widely popularized as “shovelomics” in the work of Trachsel et al. [30] using visual scoring. While the term “shovelomics” is popular, the extent of its definition is not clear and debate exists whether it only refers to methods based on root crown washing and visual scoring in maize (*Zea mays* L.) or to other protocols. Therefore, “root crown phenotyping” is proposed as less ambiguous and more broadly applicable, as defined above. Root crown phenotyping has been used to enhance the understanding of soil resource acquisition by roots of soybean (*Glycine max* L.), common bean (*Phaseolus vulgaris* L.), cowpea (*Vigna unguiculata* L.), wheat (*Triticum aestivum* L.), and maize [28, 30–36].

In order to standardize measurements and increase throughput, image-based phenotyping of crop root crowns has become the standard procedure. The unique steps of image-based phenotyping are acquiring and analyzing the image, which are of equal importance with regard to creating a reproducible and reliable protocol. The first example of image-based root crown phenotyping used a custom imaging booth with software to control vision cameras and conduct image analysis implemented in MATLAB that provided two measures, fractal dimension, and top root angle [29]. The Digital Imaging of Root Traits (DIRT) platform attempted to relax imaging requirements by allowing use of any consumer camera with roots generally placed on a dark background in less-controlled lighting conditions and currently focuses on a free cloud-based image analysis pipeline, though a Linux installation is possible [10, 37]. The cloud-based platform of DIRT requires uploading potentially thousands of root images, which is time consuming, and the less-controlled imaging protocol can lead to segmentation failures. The Root Estimator for Shovelomics Traits (REST) platform included an imaging “tent” to provide more uniform lighting, a DSLR consumer camera controlled using the manufacturer's software, and a MATLAB executable for image analysis but does not extract root length [32]. The Multi-Perspective Imaging Platform (M-PIP) includes five point-and-shoot cameras along a 90**°** arc in an imaging box, command line camera control software for Linux, and MATLAB scripts for image analysis [38]. These platforms have advanced the field of root crown phenotyping, but advances can still be made to increase access to these technologies and to optimize imaging, image analysis, and data processing.

The aim of this study was to develop a phenotyping platform for both high-throughput image acquisition and image analysis of root crowns from the field to address these gaps. The imaging hardware was designed to be ergonomic, portable, reproducible, and affordable. The imaging software was designed for rapid plant phenotyping and usability and to facilitate downstream image and data analysis. The image analysis software was designed to process images quickly, run reliably, and analyze in an automated batch mode once initial settings are provided. The imaging software and hardware were tested by acquiring thousands of root crown images in two environments, the image analysis software was validated using ground-truth data based on simulated root system images, and the integrated platform was validated for correct physical units by imaging copper wires and further tested by phenotyping wheat and soybean root crowns. Together, these developments represent an integrated solution for root crown phenotyping.

## 2. Materials and Methods

### 2.1. Experimental Design

**Figure 1:**
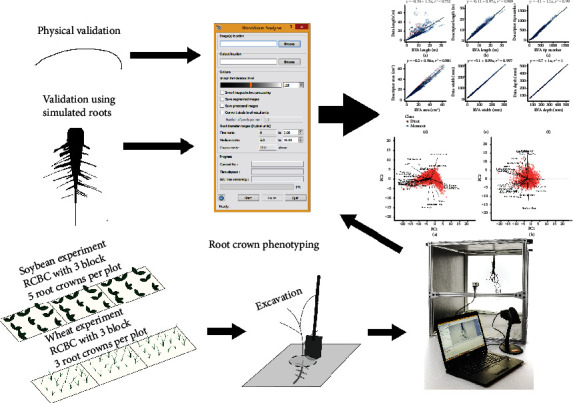
The experimental design of this study to design and validate the RhizoVision Crown platform for root crown phenotyping included constructing a hardware platform, developing a software for imaging, and developing a software for image analysis. The image analysis was validated for root measurements using simulated monocot and dicot images. The combined platform was validated for accuracy of physical measurements by imaging copper wires of known diameter. Finally, root crown phenotyping was tested using a soybean experiment in Missouri and a wheat experiment in Oklahoma.

In designing and validating a new phenotyping platform for root crowns, several related tasks and experiments were conducted (Figure 1). For validation of the image analysis software RhizoVision Analyzer, known physical and simulated calibration images were analyzed. Then, in order to test the hardware platform, imaging software, and analysis software for root crown phenotyping, root crowns for a soybean population in Missouri and a wheat population in Oklahoma were excavated and imaged using the RhizoVision hardware platform and Imager. These experiments are discussed in detail below.

### 2.2. RhizoVision Crown Hardware

**Figure 2:**
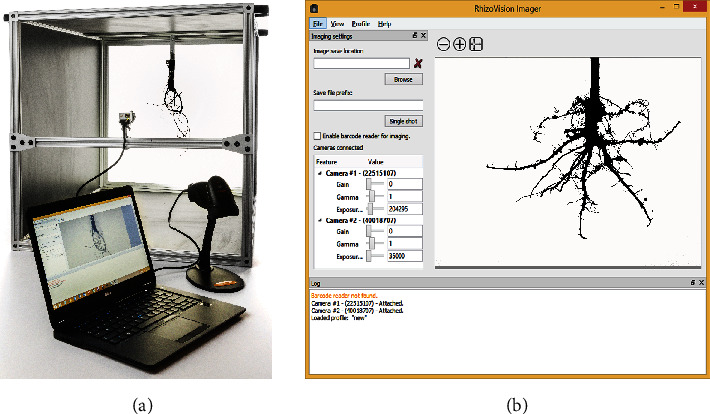
RhizoVison Crown hardware and software for root crown imaging. Root crowns are placed into the imaging unit (a) with a backlit panel for framing the root crown and a laptop connected to a vision camera and USB barcode scanner. The vision camera is controlled using the software RhizoVison Imager (b) which has a user interface for controlling camera settings, provision of live camera view, and image export settings.

The RhizoVision Crown hardware platform (Figure 2(a)) is a backlit imaging box designed to produce near binary images, where the background is near white and the foreground (root crown) is a near black silhouette. This is achieved by use of a 61cm × 61cm LED edge lit flat panel light (Anten, 40 watts, 6000 K light color) affixed with epoxy to the back of an imaging box. The imaging box is constructed from T-slotted aluminum profiles (80/20 Inc., Columbia City, IN) that were assembled to make a box measuring 65.5cm × 65.5cm × 91.4cm. Foamed black PVC panels were custom cut (TAP Plastics, Stockton, CA) and placed between profiles to isolate the interior from outside light. A root crown holder was constructed by attaching a spring clamp to the bottom of a foamed PVC panel measuring 22.86cm × 30.48cm. On the top of the root holder panel, a screen door handle was attached to assist with the placement and removal of the root holder on the instrument. Detailed images, a schematic plan, and the part list for the aluminum frame are available as Supplementary Material 1. A root crown is clamped onto the holder, and the holder panel is placed in an indentation designed into the top of the imaging box such that root crowns are consistently placed at the desired position. At one side of the imaging box is the LED panel, and on the other is a CMOS sensor monochromatic vision camera (Basler acA3800-um, Graftek Imaging, Inc., Austin, TX) using a 12 mm focal length lens (Edmund Optics 33-303, Graftek Imaging, Inc., Austin, TX). This camera faces the light panel and is focused on the root crown. The camera is connected to a laptop computer USB 3.0 port using a USB 3.0 cable (Micro-B male to A male connectors). For the recommended barcode mode, a USB barcode scanner was also connected to the laptop (Tautronics, Fremont, CA). The total cost of this platform was approximately $1,200 USD at the time of publication. The imaging software is described in the following section.

### 2.3. RhizoVision Imager

The imaging hardware is controlled by the RhizoVision Imager (Figure 2(b)). The software is open-source and can be downloaded for free from 10.5281/zenodo.2585881 (for x86_64 processor). The program can connect to multiple Basler USB 3.0 vision cameras using the Basler Pylon SDK to send commands from the laptop to the camera, capture images, and transfer image data from the camera to the laptop. A USB 3.0 cable is used both to transfer information and to supply power to the camera. For each camera, the parameters Gain, Gamma, and Exposure Time can be changed depending on experimental needs (Figure 2(b)). Using the lens mounted on the camera, the aperture and focus of the lens can be modified. In order to account for the three-dimensional shape of root crowns, small aperature settings with deeper depths of field are recommended.

The program starts with a live view for a connected camera. If multiple cameras are connected, the live view for each can be changed in the View menu. The live view can be zoomed in and out to view a specific area in the image. To start capturing images from the connected cameras, a directory location needs to be specified in which to save the images. For single shots, the user may enter an image file name. File names of all the captured images are appended by the camera number and by the number of times the image was taken with the same name and camera number. This ensures that the images are not overwritten and allows for multiple subsamples of the same biological replicate to be acquired using the same identification.

The program also includes a barcode mode for triggering image acquisition and designating file names. A barcode reader is automatically detected when connected by the user. After selecting the barcode mode (“Enable barcode reader for imaging”), scanning a barcode will acquire the image and save with a file name that includes the identification encoded by the barcode and appended with the camera and picture numbers. The program has a log window, where all the events are logged for review. This includes logging when a new image is captured, camera devices are refreshed, or a barcode scanner is attached. The camera settings can be saved as profiles in the program, which may then be reused in later experiments or modified with a text editor. The images can be saved as .BMP, .JPEG, .PNG, or .TIFF files. The RhizoVision Imager was implemented in C++ using OpenCV and uses the Basler Pylon SDK, and the user interface was developed using Qt, a cross-platform GUI toolkit. The program depends on the freely available Pylon runtime from the camera manufacturer. The RhizoVision Imager software is an open-source software with a modified GPL license, designed for Windows 10, and does not require installation.

### 2.4. RhizoVision Analyzer

**Figure 3:**
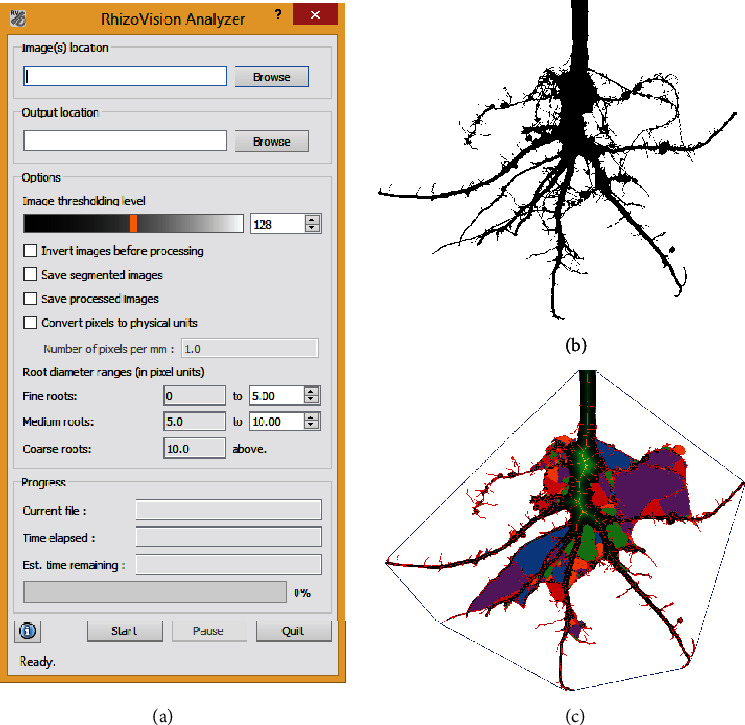
RhizoVision Analyzer for automated batch analysis of root crown images (a). The software has a user interface (a) for selecting input and output folders, choosing image threshold levels before analysis, classifying root diameter ranges, and saving options. The segmented image (b) and feature image (c) are optionally generated by the RhizoVision Analyzer. The feature image shows a blue convex polygon that is fit around the entire root system for extraction of the convex area. The boundary and skeletal pixels are shown in red and the distance transform is shown in green. The “holes” or the background image patches that were disconnected due to the overlapping of foreground pixels are randomly colored for distinction.

The RhizoVision Analyzer (Figure 3(a)) was designed to quickly analyze the images acquired using the RhizoVision Crown platform and the Imager software. The Analyzer is open-source with a modified GPL license and can be downloaded for free from 10.5281/zenodo.2585891 (for x86_64 processors). The overall goal in the design of the RhizoVision Analyzer was to create a simple-to-use and robust program that batch processes a folder containing root crown images and outputs a data file with the measures for each sample in a form convenient for data analysis. A total of 27 phenes are extracted from each input image, which are stored in a CSV text file. The Analyzer has an option to output segmented images (Figure 3(b)) as well as processed images on which visual depictions of the extracted features are drawn on the segmented image (Figure 3(c)). The program also stores a separate metadata file in CSV text format, which contains the user defined options.

**Figure 4:**
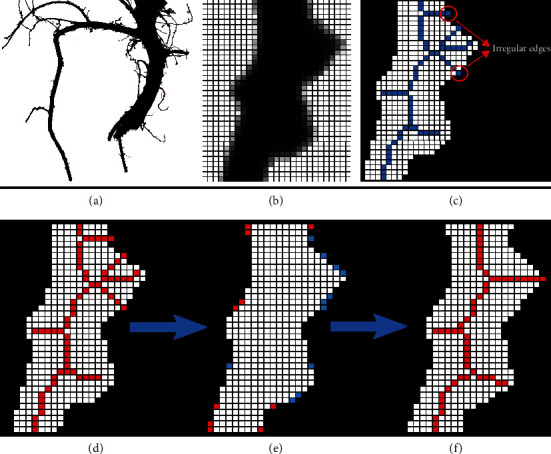
Example of how the RhizoVision Analyzer skeletonizes root crown images before extraction of measurements. A small region of interest is selected (a) and magnified (b) for demonstration purposes. The thresholded image of the region of interest shows that due to the irregular edges, the generated skeletal structure contains lateral roots that are nonexistent (shown in blue) (c). The skeletal structure of the root is then smoothed to reduce falsely classified lateral roots before line smoothing operation (d). During the line smoothing operation, pixels are either added (shown in red) or deleted (shown in blue) (e). Finally, the skeletal structure of the root after the line smoothing operation has the falsely classified lateral roots removed (f).

Coupled with the optimized image acquisition using the hardware platform, segmentation of the root crown images from the background requires only thresholding of the greyscale values for each pixel with minimal loss of data (Figure 4(b)). Thresholded (binary) or greyscale images from other platforms may also be used. The input image may have irregular edges that lead to nonexistent skeletal structures being created (Figure 4(c)). In order to address this, the edges of the input image are smoothed using the Ramer-Douglas-Peucker algorithm [39, 40]. According to the algorithm, the contour is simplified into piecewise connected straight lines where each straight line is an approximation of the original contour points within a specified pixel distance threshold from the line approximation. The algorithm starts by connecting the start and end points of a contour with a straight line and computes the maximum distance between the pixels on the contour to the line. If this distance is more than the threshold of 2 pixels, then the line is split at that pixel where the maximum distance is found and a piecewise connection of straight line is created. The algorithm then proceeds recursively as described above for each piecewise straight line. In the RhizoVision Analyzer, the distance threshold parameter was set to be 2 pixels because, given the object size using this hardware configuration, the irregular edges spanned about 2 pixels and simplifying the contours using this threshold did not result in great changes in the segmented image but led to substantial reduction in the number of erroneous lateral roots. Any decrease in the threshold may increase nonexistent lateral roots, and any increase may lead to oversimplification of the root topology that adversely affects the extracted phenes. After this procedure, the overall shape of a root segment does not change substantially, but the skeletal structure is simpler and has fewer nonexistent lateral roots (Figures 4(d)–4(f)). This procedure will not lead to a completely error-free skeleton in all situations, which is a common problem across all root image analysis software, and no manual correction is possible. Pruning of these extra short root skeletal segments is another option to be explored in the future.

**Figure 5:**
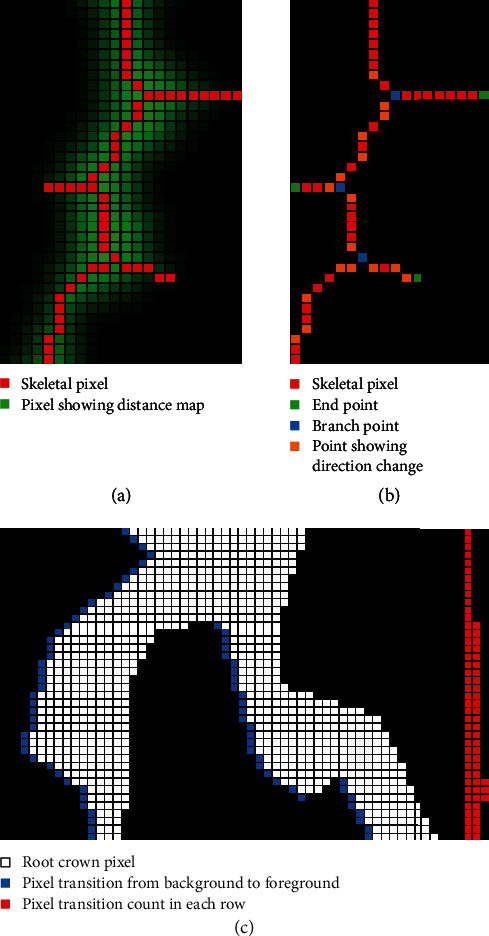
Example of how the RhizoVision Analyzer extracts quantitative features from the root crown image. For each pixel within the root crown skeleton, the corresponding value from the distance map is used to estimate root diameter (a). Topological information is extracted from the skeletal structure such as branch points (shown in blue), root direction change (shown in orange), and end points (shown in green) (b). Finally, for the root counting procedure (c), a pixel transition is marked in a horizontal line scanning operation (shown in blue) for each row and is recorded for counting the number of roots in that row (shown in red). This profile is then sorted to extract the median and maximum number of roots.

On each row of the segmented and smoothed image, a horizontal line scanning operation is performed from the left to the right side of the image, where each pixel transition from background to foreground (plant root pixel) is counted. These pixel transitions form a plant root count profile along the depth of the root crown, from which the median and the maximum number of roots are determined (Figure 5). Since the root crown is hung vertically using the clamp at the top, root segments will be under the influence of gravity and less rigid roots may fully collapse, which will prevent accurate counting. Roots may also be lost during excavation and washing. However, as the purpose of phenotyping is generally uncovering relative differences among genotypes, this method is sufficient while imperfect. While the number of roots in each row of the image may be susceptible to the slight angle variations at which the root is held, by determining the median and maximum of the number of roots, the extracted phenes are ensured to be relatively stable. The network area of the image is determined by counting the total number of plant root pixels in the image. Further, a convex polygon is fit on the image and the area is noted as a convex area.

A precise distance transform is computed on the line smoothed image in order to identify the medial axis. The distance transform [41] of an image is the map of distance of each pixel to the nearest background pixel. The distance metric used here is the Euclidean distance metric (Figure 5(a)). The medial axis is a set of loci on the distance transform that are equidistant from at least two background pixels and is identified from the ridges formed on the distance transform map (Figure 5(a)). In order to make a fully connected skeletal structure, additional pixels are added using the connectivity preserving condition from the Guo-Hall thinning algorithm [42, 43] and the end points of the ridges are connected using the steepest accent algorithm. The contours of the segmented image are identified for determining the perimeter of the plant root structure.

Using the generated skeletal structure, topological properties such as the branch points and end points are identified (Figure 5(b)). The skeletal pixels connecting one branch point to another branch or end point are identified as root segments. This part was then validated to ensure that the root skeletal structure reconstructed from the root segments replicates the original skeletal structure. Rules were also designed so that the end points were used only once for any root segment, and each branch point is used three times either as a start or end point of a root segment. The number of end points is noted as the number of root tips. For each skeletal pixel in every root segment, a 40 × 40 neighborhood window is selected. Within each window, the slope of the principal component of the skeletal pixels is computed and the average angle is determined using these slopes. The window size of 40 × 40 is selected as it is large enough so that smaller differences in the angle are ignored giving more importance to larger changes in direction. Using these angles, the numbers of shallow-angled, medium-angled, and steep-roots in an image are noted as histogram bins, by grouping the computed angles in ranges of 0° to 30°, 30° to 60°, and 60° to 90° from the horizontal, respectively. This histogram is normalized and the bins are named as shallow, medium, and steep angle frequencies. Further, an average of all the angles is computed and noted as average root orientation. A similar normalized histogram is constructed using the skeletal pixels on the root diameter. The three histogram bins are allowed for the user to be specified from the user interface of the RhizoVision Analyzer. These bins are noted as fine, medium, and coarse diameter frequencies. Also, the average, median, and maximum diameters are identified from the diameters of all the skeletal pixels. The plant root area below the pixel having the maximum diameter is noted as the lower root area. The segmented and edge-smoothed image is color inverted, connected component analysis is performed to count the number of holes, and an average of all the hole areas is computed to determine the average hole size.  Table 1 briefly describes the list of features extracted from the root crown images.

The RhizoVision Analyzer is implemented in C++ using the OpenCV library. The user interface of the program is developed in Qt, a cross-platform GUI toolkit. The program can utilize a CPU's vectorization facilities using Intel's AVX 2.0 technology, to execute the algorithms faster on newer computers. All pixel-based measures are converted to appropriate physical units if the user supplies the number of pixels per millimeter before analysis. Depending on the exact computer system, the Analyzer can be expected to routinely process each image in a fraction of a second.

### 2.5. Validation Using Copper Wire and Simulated Root Systems

In order to validate the ability of the RhizoVision Analyzer to correctly determine physical measurement units from pixel-based analysis of images, copper wires of different diameter gauges were imaged and analyzed. The gauges (American Wire Gauge, AWG) used were 10, 16, 22, 28, and 32 which represent a diameter range of 0.20–2.57 mm. Two lengths of wire were used for each gauge for a total of ten. The ground-truth diameter was measured using a micrometer. The ten wires were imaged individually using the RhizoVision Crown hardware. The pixel to millimeter conversion was determined from the manually determined pixel width of a coin envelope imaged with the hardware platform and which was manually measured using a micrometer. These wire images were processed using the RhizoVision Analyzer with 13.63866 used to scale from pixels to millimeters.

To validate the diverse root measures generated by the RhizoVision Analyzer software, 10,464 simulated images of dicot and monocot root systems from Lobet et al. [44] were processed (total elapsed time of 1 hour 7 mins on an Intel processor with 8 cores, 3.7 GHz of clock frequency, and 16 GB of RAM memory). Lobet et al. [44] define the ground-truth data as the known measurements from the three-dimensional simulations and the descriptor data as those derived from projected 2D images using RIA-J image analysis. Common features from the RhizoVision Analyzer and RIA-J include length, area, tip number, width, and depth.

### 2.6. Field Sites and Root Crown Phenotyping

#### 2.6.1. Phenotyping Soybeans in Missouri

A F_5_-derived soybean recombinant inbred line (RIL) population derived from a cross of PI398823 × PI567758 was planted at the Bradford Research Center near Columbia, MO, on a Mexico silt loam soil (fine, smectitic, and mesic Aeric Vertic Epiaqualf). The parental lines of this population were identified to differ in root crown architecture based on the characterization of a soybean diversity panel prior to the creation or use of this platform (F.B. Fritschi, unpublished). Preplanting soil tests indicated that no P or K fertilizer application was necessary. Prior to sowing, the seedbed was prepared by one pass with a disc to approximately 0.15 m depth, which was followed by a pass with a harrow. The 185 RILs and two parental lines were sown in a randomized complete block design with three replications on 14 May 2017 at a density of 344,000 plants ha^−1^ in single 3 m long rows with a row spacing of 0.76 m. Weed control consisted of a preplant burn-down application of glyphosate (0.73 kg ha^−1^ a.i.) and postplanting applications of acetochlor (0.6 kg ha^−1^ a.i.), bentazone (0.27 kg ha^−1^ a.i.), and clethodim (0.03 kg ha^−1^ a.i.), and these herbicide applications were supplemented by manual weeding as needed. Additionally, two applications of zeta-cypermethrin S-cyano (0.1 kg ha^−1^ a.i.) were conducted to control insects.

Five root crowns for each plot were excavated at the beginning of the R6 stage the week of 21 September 2017 using a shovel. For each focal plant, the shovel was inserted such that the width of the blade was parallel to the row and midway between two rows on each side of the plant. The blade was inserted as deeply as possible, and on the second insertion, the shovel was leveraged in order to pry up the plant. The soil was very loose so the root crowns only needed shaking to remove the majority of soil and were not washed. The root crowns were imaged using the RhizoVision Crown in the field using a gasoline electric generator for power. The lens of the camera was placed at a working distance of 56 cm from the center of the root crown (the bottom of the clamp) for a resolution of 12.7787 pixels per mm. The lens aperture was set to f/11.0 to maximize the depth of field to accommodate the 3D root crown. The exposure time was set to 14 ms and gamma was set to 3.9 in order to optimize contrast. Roots were placed in the orientation that appeared as the widest to the user in order to standardize measurements. A total of 2,799 images were acquired from wheat root crowns grown in Missouri.

#### 2.6.2. Phenotyping Wheat in Oklahoma

The wheat population is a recombinant inbred line (RIL) population with 184 F_5:7_ lines derived from a cross between TAM111 × TX05A001822. The population was created for mapping QTL or genes contributing to a number of important agronomic traits, and root phenes were not previously evaluated. “TAM 111” is one of the most planted hard red winter wheat cultivars in the Southern High Plains and has adapted to both dryland and irrigated conditions [45] while TX05A001822 is an advanced breeding line with superior bread making quality from the Texas A&M AgriLife Research.

The population was planted in a randomized complete block design with three replications of 1.5 m wide by 0.9 m long plots with seven rows and seeded at a rate of 148 kg ha^−1^ on 11 November 2017 at Burneyville, Oklahoma. The field was clean tilled prior to planting and rain-fed with no supplemental irrigation. Fertilization was first preplant incorporated with 56 kg ha^−1^ nitrogen and then top-dressed with 56 kg ha^−1^ nitrogen on 23 January 2018 based upon rainfall. Phosphorous and potassium concentrations were sufficient based on soil test results prior to planting. Weeds were controlled with 247 kg ha^−1^ of glyphosate at planting and 0.02 kg ha^−1^ of Glean XP at Zadoks growth stage 13. Postemergence application of 1.12 kg ha^−1^of 2,4-D was used on 14 February 2018 for broadleaf weed control.

Root crowns were excavated near grain maturity on 14–15 May 2018. Several plants were harvested with a single excavation because of the high population density. The shovel was inserted parallel to the row with its back against the neighboring rows on each side of the focal plants, and a whole group of plants was lifted out then placed into a large plastic bag with a barcode label affixed for sample identification. These bags were taken to the washing station where the group of root crowns in soil were placed in water with dish soap and allowed to soak in one of 20 plastic bins. After soaking and gently moving back and forth in water to remove most soil, the root crowns were removed and washed with a water hose spray nozzle with light pressure for a few seconds to clean more thoroughly. The group of plants remained together and were placed back into the plastic bags. These bags were transported back to the lab and kept in a cold room for one week while imaging using the RhizoVision Crown. Three plants were selected from the group, and the barcodes were used for triggering image acquisition and saving file names. The lens of the camera was placed at a working distance of 51.5 cm from the center of the root crown (the bottom of the clamp) for a resolution of 14.0315 mm per pixel. The lens aperture was adjusted to f/11.0. Exposure time and gamma were set to 14 ms and 3.9, respectively. A total of 1,753 images were acquired from wheat root crowns grown in Oklahoma.

### 2.7. Statistical Analysis

Statistical analyses were employed using R version 3.5.1 (R [46]) through RStudio version 1.1.45 [47]. Linear regressions were fit using the “lm” function. Principal component analysis was conducted using the “prcomp” function after scaling and centering the data. The R package “reshape2” [48] was used to format the data before plotting. The R package “ggplot2” [49] was used for data visualization. Other packages used included “dplyr,” “purr,” and “patchwork.” Broad-sense heritability was calculated based on Falconer and Mackay [50] as

(1) $$\begin{array}{c} H^{2}=\frac{\sigma_{g}^{2}}{\sigma_{g}^{2}+\left(\sigma_{e}^{2}/r\right)}. \end{array}$$

The variables *σ*_*g*_^2^, *σ*_*e*_^2^, and *r* represent the variance of the genotype effect, variance of the local environment effect, and the number of replicates (blocks), respectively. The variances were obtained by fitting a mixed model including genotype as a random effect and block as a fixed effect using the lme4 package [51]. The data for the five root crowns of soybean and three root crowns of wheat from each plot were averaged before subsequent computation of heritability. The heritability computed using the equation above was plot-based, whereas the correlations were computed using plant-based data. All statistical code and data files needed are available to download at 10.5281/zenodo.3380473.

## 3. Results

### 3.1. Physical Calibration

**Figure 6:**
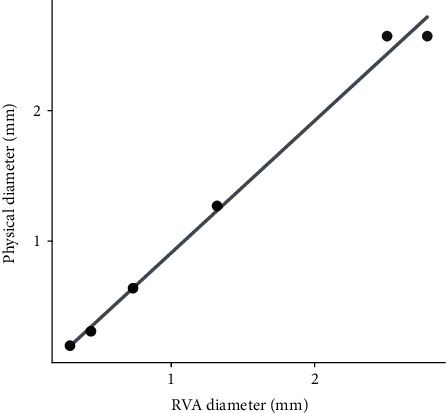
Regression between diameters extracted using the RhizoVision Analyzer of copper wires imaged with the camera-based hardware and caliper-measured diameters (each diameter has two points).

In order to ensure that the correct physical units can be determined by the integrated platform, copper wires of known diameters ranging from 0.20 to 2.57 mm were imaged with the RhizoVision Crown hardware, and the correct pixels per mm conversion factor was supplied to the Analyzer for analysis. Regression of the computed diameters versus caliper-measured diameters showed nearly exact correspondence (*y* = −0.1 + 1.01*x*, *R*^2^ = 0.99, *p* < 0.01), which indicates the physical units provided by the Analyzer are accurate when the user supplies the pixels to mm conversion (Figure 6).

### 3.2. Validation Using Simulated Root System Images

**Figure 7:**
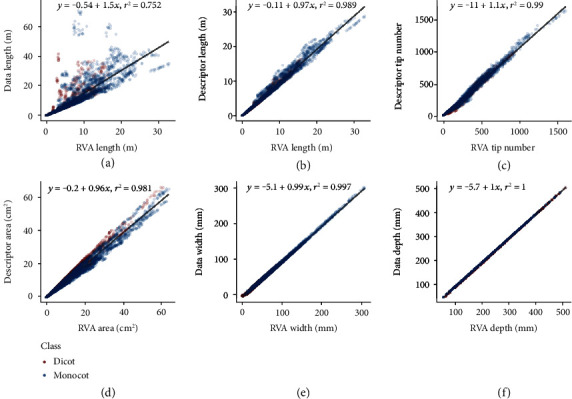
Values of coefficient of determination and linear fit equations between root features extracted using the RhizoVision Analyzer (RVA) and ground-truth-simulated root data or published original descriptors. Scatter plots include linear regressions of (a) RVA length against ground-truth data length, (b) RVA length against descriptor length, (c) RVA tip number against descriptor tip number, (d) RVA area against descriptor area, (e) RVA width against descriptor width, and (f) RVA depth against descriptor depth.

In order to validate the root measures from the RhizoVision Analyzer, a publicly available dataset consisting of more than 10,000 simulated root system images was utilized from Lobet et al. [44], which include ground-truth data from 3D models and descriptors from analysis of the 2D projections analyzed using RIA-J root image analysis software. The ground-truth total root length was underestimated by the Analyzer (*y* = −0.54 + 1.55*x*, *R*^2^ = 0.75, *p* < 0.01) (Figure 7(a)), which is to be expected because the original simulated roots were three-dimensional but the processed images are projected to two dimensions. The RIA-J extracted descriptor length provided was similar to the Analyzer length (*y* = −0.11 + 0.97*x*, *R*^2^ = 0.99, *p* < 0.01) (Figure 7(b)), indicating that the Analyzer performs similarly to the previously used software. Tip number (*y* = −11.49 + 1.07*x*, *R*^2^ = 0.99, *p* < 0.01) (Figure 4(c)), root crown area (*y* = 0.20 + 0.96*x*, *R*^2^ = 0.98, *p* < 0.01) (Figure 7(d)), root crown maximum width (*y* = −5.07 + 0.99*x*, *R*^2^ = 0.99, *p* < 0.01) (Figure 7(e)), and root crown maximum depth (*y* = −5.68 + 1.00*x*, *R*^2^ = 1.00, *p* < 0.01) (Figure 7(f)) all indicate that the Analyzer extracts phenes that have the same physical units (slopes equal one) and that account for a high proportion of variance of the phenes extracted by RIA-J software.

### 3.3. Phenotyping Soybean and Wheat Root Crowns

In order to validate the entire hardware and software platform, 2,799 of soybean root crowns of 187 lines grown in Missouri and 1,753 images were acquired of wheat root crowns of 186 lines grown in Oklahoma. For both species, all of the root crowns were imaged with the hardware platform using the RhizoVision Imager and processed by the RhizoVision Analyzer with no segmentation failures, indicating the hardware provides reproducible images that are optimized for image analysis irrespective of plant species. On a computer with an 8-core Intel processor with 16 GB of RAM, analysis of the 2,799 soybean images took 17 minutes and the 1,753 wheat images took 11 minutes.

**Figure 8:**
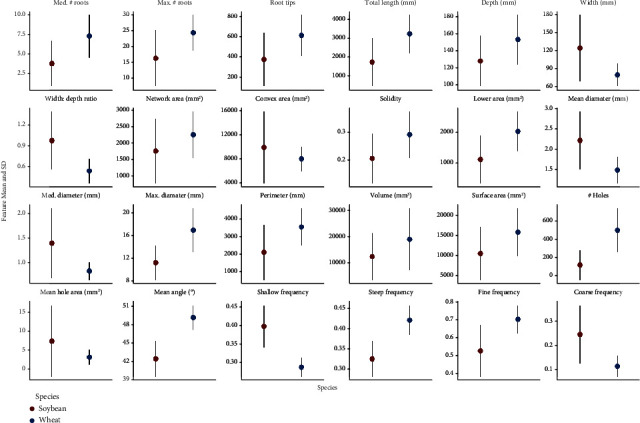
Summary of means and standard deviations of various features extracted from soybean (*n* = 2,799) and wheat (*n* = 1,753) root crown images using the RhizoVision Crown platform.

The means and standard deviations were computed for the extracted phenes (defined in Table 1) independently for the wheat and soybean populations (Figure 8) grown at the two different sites. The average total root length of soybean root crowns was 1.70 ± 1.29m, number of root tips was 368.27 ± 264.47, maximum width was 123.09 ± 55.54mm, and the depth of the roots was 127.36 ± 29.73mm. In general, the entire root crown fit within the field of view of the camera so width and depth measurements are accurate. The soybean root crowns showed solidity values of 0.21 ± 0.09, the median root diameter of 1.40 ± 0.71mm, the hole number of 118.66 ± 163.76, and the average hole size of 7.52 ± 9.47mm2. Finally, the average root orientation of every pixel in the skeletal structure of the soybean root was 42.50° + 2.94° from the horizontal. The average total root length of wheat root crowns was 3.20 ± 1.02m, number of root tips was 606.77 ± 204.58, maximum width was 78.54 ± 18.83mm, depth of the roots was 152.56 ± 29.21mm, solidity was 0.29 ± 0.08, median root diameter was 0.83 ± 0.18mm, hole number was 500.27 ± 240.54, hole size was 3.24 ± 1.93mm2, and average root orientation of every pixel in the skeletal structure of the wheat root was 49.22° + 2° from the horizontal. The parents of the soybean population differed substantially for a majority of the phenes, but the parents of the wheat population did not differ (Supplementary Material 2).

**Figure 9:**
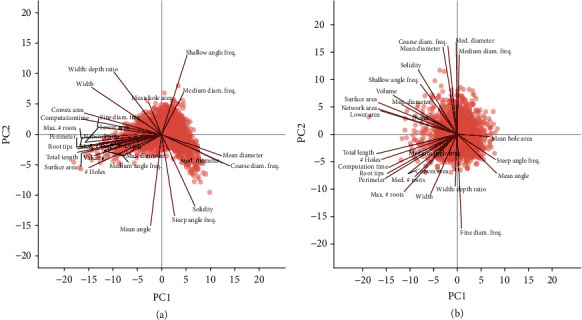
Principal component analysis of root crown phenes from the soybean (a) and wheat (b) datasets. Points represent the scores of principal components 1 and 2 (PC1 and PC2, respectively) for each species. Labelled lines demonstrate the correlation of phenes to principal component scores.

Principal component analysis was used to identify the major linear phene combinations that maximize the multivariate variation (Figure 9, Supplementary Material 3). Principal components (PC) 1 and 2 explained 51.94% and 12.97% of the multivariate variation, respectively, for the phenes extracted for soybean root crowns (Figure 9(a)). The phenes that loaded most strongly onto PC1 were size-related phenes such as total root length, perimeter, number of root tips, number of holes, several measures of root areas, and some contribution from diameter measures. PC2 was dominated by the mean angle and angle frequencies. PCA of wheat root crowns (Figure 9(b)) showed that the PC1 and PC2 explained 34.99% and 27.02% of the multivariate variation, respectively. The phenes that loaded onto PC1 were size-related phenes such as total root length, perimeter, number of root tips, number of holes, and maximum diameter. PC2 was strongly dominated by median diameter and the diameter frequencies.

**Figure 10:**
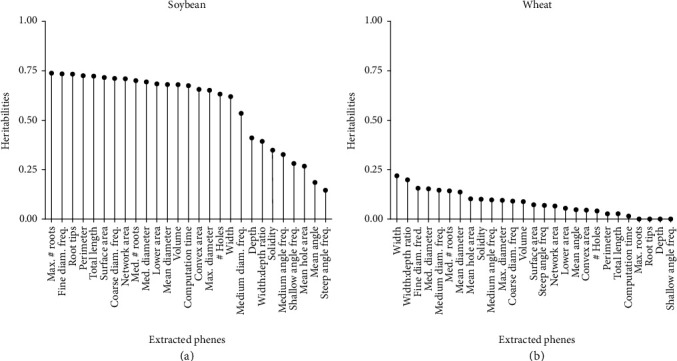
Heritabilities of each phene extracted using the RhizoVision Analyzer for soybean (a) and wheat (b) datasets.

In order to evaluate the possibility to use these root phenes for breeding, broad-sense heritabilities were computed for the phenes extracted from soybean root crown images (Figure 10(a)). In soybean, a majority of the phenes had heritabilities greater than 0.50. The maximum heritability was observed with maximum number of roots at 0.74. The phenes with lower heritabilities were the ratios, mean angle, and orientation frequencies. Heritabilities for the wheat root crowns were generally lower, ranging from 0 to 0.22 for the maximum width (Figure 10(b)).

## 4. Discussion

Over the past few years, the throughput, reliability, and standardization of root crown phenotyping has increased using digital imaging and image-based analysis software, such as DIRT [37], REST [32], and M-PIP [38]. Minimizing cost, increasing throughput, and improving reliability are key demands for developing high-throughput root phenotyping platforms. The integration of the RhizoVision Imager software with the RhizoVision Crown hardware platform facilitates phenotyping with the end-user in mind by utilizing a backlit approach and a simple clip-and-replace system for replacing root crowns.

The RhizoVision Imager allows live view so that the user may verify that the images are high-contrast and framed correctly, stores camera settings, and has a barcode scanning mode that saves images with the sample identification. However, the use of RhizoVision Imager is not necessary to successfully use the hardware platform or the image analysis software. Many machine vision and consumer point-and-shoot camera manufacturers, including Basler with Pylon Viewer, provide imaging software limited to use with their cameras. For DSLR consumer cameras, digiCamControl (free, open-source) and CaptureGrid (commercial) provide control of cameras from several major brands. The main advantages to using the RhizoVision Imager are the barcode triggering mode that facilitates sample tracking and the relatively simple user interface. The Pylon API is based on a vendor-independent international standard called GenICam, so extending the Imager for more general use may be possible. While a color camera could also be used with this platform, monochrome cameras produce sharper images with a backlight. The ergonomics evident in the hardware and control software facilitate high-throughput image acquisition.

The features extracted by the RhizoVision Analyzer were extensively validated with 10,464 simulated images of dicot and monocot root systems from a publicly available dataset. Excellent agreements were observed between root phenes like length, tip number, root crown area, root crown maximum width, and root crown maximum depth extracted using the Analyzer and published data of the simulated images. In order to validate the physical unit accuracy of the combined hardware and software, copper wires representing a range of diameters observed in roots were imaged and analyzed. In order to test the practical use of this platform, thousands of excavated soybean and wheat root crowns were phenotyped from the field.

The soybean and wheat experiments occurred at different times and at different sites, so a direct statistical comparison is not possible. However, in both experiments, root crown phenotyping occurred after flowering so root crowns were mature. Therefore, the differences observed between the species may be representative of the intrinsic differences. For example, the mean and median root diameters of root crowns are smaller for wheat compared to soybean as would be expected. Wheat root crowns are also typically less wide and with steeper angles due to the growth of nodal roots as opposed to the shallow angles of first-order laterals in soybean. While the heritabilities of features for soybean were typically greater than 0.50 with a maximum of 0.74, the maximum observed for wheat was only 0.22. Possibly, this indicates that intrinsic differences between the species make the wheat root crown less suitable for phenotyping using this method. For example, the smaller diameter wheat roots are more flexible and when suspended orient downwards, and so differences among genotypes may be obscured. However, root crown phenotyping of field-excavated wheat root crowns was previously used to confirm shallow and steep angles of lines measured in a lab-based seedling screen with success [33], which indicates that the lower heritabilities observed here may not be due to an inherent incompatibility of the method. Another explanation for low heritability is simply that there is not substantial genetic variation for these root phenes present in the RIL population used which is possible because the wheat parents were not selected based on root characteristics, while the soybean parents were selected based on contrasting root system architecture prior to the creation of this platform. Based on the data collected with this platform, the soybean parents were confirmed to differ substantially for many of the measured phenes, while the wheat parents did not. Still, even in the absence of parental differences, progeny can exhibit diversity due to transgressive segregation so the results are not conclusive. For species with more flexible roots, refinements to the protocol such as laying the root crown on a flat surface rather than suspending and including more sup-replicates from each plot should be investigated. The imaging box described here could easily be oriented to have the backlight facing up for this use. Additional image-based measures could further improve plant classification and characterization of root topology, for example, extracting new root phenes such as lateral root branching density or angles and lengths of specific classes of roots through optimized algorithms. Incorporation of morphometric descriptors [52] could simplify representation of data, such as persistent homology [53].

In conclusion, RhizoVision Crown is a cost-effective and high-throughput platform that has the potential to increase access to technologies for root crown phenotyping. The platform builds upon previous platforms [29, 32, 37, 38] by optimizing image acquisition using a backlight and the barcode option, using custom imaging software designed for phenotyping, and using image analysis software with a simple graphical interface designed for batch processing. All software are free and ready-to-use on Windows 10. The platform has been validated using ground-truth measures of a simulated dataset and successfully extracted root phenes from field-excavated root crowns of a cereal and a legume species. The ergonomics of use, the integration of all hardware and software, and the extensive validation tests serve as a benchmark for other plant phenotyping platforms [54]. This technology will increase access to root crown phenotyping as a method to acquire data for functional phenomics [55], genetic mapping, use in breeding programs, and understanding how root phenes can address agricultural unsustainability and food insecurity.

## Figures and Tables

**Table 1:** The list of 27 features extracted from each root crown image by the RhizoVision Analyzer.

Features extracted	Description
Median and maximum number of roots	The number of roots is counted by performing horizontal line scans from left to right in each row through the segmented image. In each of the line scan, pixel value transitions are checked from the previous pixel value to the current pixel value on its right side. If the current pixel value changes from 0 to 1, a root is recorded. The number of roots is recorded from each row of the segmented image, and the median and maximum numbers of roots are determined from these values.
Number of root tips	Count of the total number of tip pixels in the skeletonized image.
Total root length	Computed by determining the total Euclidean distance of medial axis pixels in the skeletonized image.
Depth, maximum width, and width-to-depth ratio	The maximum depth and maximum width of the root in the segmented image. The ratio of maximum width to depth of the image is noted as the width-to-depth ratio.
Network area, convex area, and solidity	The total number of pixels in the segmented image. The convex hull of the segmented image is the minimal-sized convex polygon that can contain the root. From this convex hull, the convex area is determined. The ratio of the network area and the convex area is given as the solidity.
Perimeter	Perimeter is the total Euclidean distance of contour pixels in the segmented image.
Average, median, and maximum diameters	For each pixel in the skeletonized image, the distance to the nearest nonroot pixel is computed. This distance is used as a radius to fit a circle. The diameter of the circle at each pixel is noted as the root diameter. The list of diameters from all the medial axis pixels is used to determine the average, median, and maximum diameters for the entire root crown.
Volume and surface area	Using the radii determined earlier, the sum of all cross-sectional areas across all the medial axis pixels is noted as the volume and the sum of the perimeter across all the medial axis pixels is noted as the surface area.
Lower root area	The lower root area is the area of the segmented image pixels that are located below the location of the medial axis pixel that has the maximum radius.
Holes and average hole size	Holes are the disconnected background components and indicative of root branching and complexity. They can be counted (number) by inverting the segmented image. The average hole size (area) is also calculated.
Average root orientation	For every medial axis pixel, the orientation at the pixel is computed by determining the mean orientation of medial axis pixels in a 40 × 40 pixel locality. The average of all these orientations is noted as the average root orientation.
Fine, medium, and coarse diameter frequencies	From the skeletal image, the medial axis pixels are grouped into fine or coarse roots based on the diameter values at the pixels.
Shallow, medium, and steep angle frequencies	Given the skeletal image, for every pixel in the medial axis, the locations of the medial axis pixels in a 40 × 40 pixel locality are used to determine the orientation of these pixels in the locality. This orientation is noted for every medial axis pixel. These orientation frequencies are grouped in bins less than 30, less than 60, and less than 90 degrees from the horizontal.
Computational time	The time taken to extract features for one image.

## Supplementary Materials

Supplementary Materials Supplementary Material 1 is included as a PDF containing images of the completed hardware platform from different angles, schematic drawings, and a part list for the aluminum structure. A table of the means of all extracted features for the four wheat and soybean parents is found in Supplementary Material 2. A table containing the contribution rates, or proportions of variance, from each principal component and the loadings of all features onto the components for both species is found in Supplementary Material 3.

## References

[1] Lynch J. (1995). Root architecture and plant productivity. *Plant Physiology*.

[2] Meister R., Rajani M., Ruzicka D., Schachtman D. P. (2014). Challenges of modifying root traits in crops for agriculture. *Trends in Plant Science*.

[3] Rogers E. D., Benfey P. N. (2015). Regulation of plant root system architecture: implications for crop advancement. *Current Opinion in Biotechnology*.

[4] York L. M., Nord E. A., Lynch J. P. (2013). Integration of root phenes for soil resource acquisition. *Frontiers in Plant Science*.

[5] Lynch J. P. (2011). Root phenes for enhanced soil exploration and phosphorus acquisition: tools for future crops. *Plant Physiology*.

[6] Pieruschka R., Poorter H. (2012). Phenotyping plants: genes, phenes and machines. *Functional Plant Biology*.

[7] Bishopp A., Lynch J. P. (2015). The hidden half of crop yields. *Nature Plants*.

[8] Eshel A., Beeckman T. (2013). *Plant Roots: The Hidden Half*.

[9] Topp C. N., Bray A. L., Ellis N. A., Liu Z. (2016). How can we harness quantitative genetic variation in crop root systems for agricultural improvement?. *Journal of Integrative Plant Biology*.

[10] Das A., Schneider H., Burridge J. (2015). Digital imaging of root traits (DIRT): a high-throughput computing and collaboration platform for field-based root phenomics. *Plant Methods*.

[11] Kuijken R. C., van Eeuwijk F. A., Marcelis L. F., Bouwmeester H. J. (2015). Root phenotyping: from component trait in the lab to breeding. *Journal of Experimental Botany*.

[12] Weaver J. E. (1925). Investigations on the root habits of plants. *American Journal of Botany*.

[13] Weaver J. E., Bruner W. E. (1926). *Root Development of Field Crops*.

[14] Böhm W. (2012). *Methods of Studying Root Systems*.

[15] Stoeckeler J. H., Kluender W. A. (1938). The hydraulic method of excavating the root systems of plants. *Ecology*.

[16] Kosola K. R., Workmaster B. A. A., Busse J. S., Gilman J. H. (2007). Sampling damage to tree fine roots: comparing air excavation and hydropneumatic elutriation. *HortScience*.

[17] Smucker A. J. M., McBurney S. L., Srivastava A. K. (1982). Quantitative separation of roots from compacted soil profiles by the hydropneumatic elutriation system 1. *Agronomy Journal*.

[18] Johnson M. G., Tingey D. T., Phillips D. L., Storm M. J. (2001). Advancing fine root research with minirhizotrons. *Environmental and Experimental Botany*.

[19] Wasson A., Bischof L., Zwart A., Watt M. (2016). A portable fluorescence spectroscopy imaging system for automated root phenotyping in soil cores in the field. *Journal of Experimental Botany*.

[20] Garré S., Coteur I., Wongleecharoen C. (2013). Can we use electrical resistivity tomography to measure root zone dynamics in fields with multiple crops?. *Procedia Environmental Sciences*.

[21] Liu X., Dong X., Xue Q. (2018). Ground penetrating radar (GPR) detects fine roots of agricultural crops in the field. *Plant and Soil*.

[22] York L. M., Ristova D., Barbez E. (2018). Phenotyping crop root crowns: general guidance and specific protocols for maize, wheat, and soybean. *Root Development: Methods and Protocols*.

[23] Beentje H. J. (2010). *The Kew Plant Glossary: An Illustrated Dictionary of Plant Terms*.

[24] Gao Y., Lynch J. P. (2016). Reduced crown root number improves water acquisition under water deficit stress in maize (Zea mays L.). *Journal of Experimental Botany*.

[25] Slack S., York L. M., Roghazai Y., Lynch J., Bennett M., Foulkes J. (2018). *Wheat shovelomics II: revealing relationships between root crown traits and crop growth*.

[26] Trachsel S., Kaeppler S. M., Brown K. M., Lynch J. P. (2013). Maize root growth angles become steeper under low N conditions. *Field Crops Research*.

[27] Wasson A. P., Richards R., Chatrath R. (2012). Traits and selection strategies to improve root systems and water uptake in water-limited wheat crops. *Journal of Experimental Botany*.

[28] York L. M., Galindo-Castaneda T., Schussler J. R., Lynch J. P. (2015). Evolution of US maize (Zea mays L.) root architectural and anatomical phenes over the past 100 years corresponds to increased tolerance of nitrogen stress. *Journal of Experimental Botany*.

[29] Grift T. E., Novais J., Bohn M. (2011). High-throughput phenotyping technology for maize roots. *Biosystems Engineering*.

[30] Trachsel S., Kaeppler S. M., Brown K. M., Lynch J. P. (2011). Shovelomics: high throughput phenotyping of maize (Zea mays L.) root architecture in the field. *Plant and Soil*.

[31] Burridge J., Jochua C. N., Bucksch A., Lynch J. P. (2016). Legume shovelomics: High–Throughput phenotyping of common bean (Phaseolus vulgaris L.) and cowpea (Vigna unguiculata subsp, unguiculata) root architecture in the field. *Field Crops Research*.

[32] Colombi T., Kirchgessner N., Le Marié C. A., York L. M., Lynch J. P., Hund A. (2015). Next generation shovelomics: set up a tent and REST. *Plant and Soil*.

[33] Maccaferri M., El-Feki W., Nazemi G. (2016). Prioritizing quantitative trait loci for root system architecture in tetraploid wheat. *Journal of Experimental Botany*.

[34] York L. M., Lynch J. P. (2015). Intensive field phenotyping of maize (Zea mays L.) root crowns identifies phenes and phene integration associated with plant growth and nitrogen acquisition. *Journal of Experimental Botany*.

[35] York L. M., Slack S., Bennett M. J., Foulkes M. J. (2018). *Wheat shovelomics I: a field phenotyping approach for characterising the structure and function of root systems in tillering species*.

[36] Le Marié C. A., York L. M., Strigens A. (2019). Shovelomics root traits assessed on the EURoot maize panel are highly heritable across environments but show low genotype-by-nitrogen interaction. *Euphytica*.

[37] Bucksch A., Burridge J., York L. M. (2014). Image-based high-throughput field phenotyping of crop roots. *Plant Physiology*.

[38] Seethepalli A., York L. M., Almtarfi H., Fritschi F. B., Zare A. (2018). *A novel multi-perspective imaging platform (M-PIP) for phenotyping soybean root crowns in the field increases throughput and separation ability of genotype root properties*.

[39] Douglas D. H., Peucker T. K. (1973). Algorithms for the reduction of the number of points required to represent a digitized line or its caricature. *Cartographica*.

[40] Ramer U. (1972). An iterative procedure for the polygonal approximation of plane curves. *Computer Graphics and Image Processing*.

[41] Felzenszwalb P. F., Huttenlocher D. P. (2012). Distance transforms of sampled functions. *Theory of Computing*.

[42] Guo Z., Hall R. W. (1989). Parallel thinning with two-subiteration algorithms. *Communications of the ACM*.

[43] Lam L., Lee S.-W., Suen C. Y. (1992). Thinning methodologies-a comprehensive survey. *IEEE Transactions on Pattern Analysis and Machine Intelligence*.

[44] Lobet G., Koevoets I. T., Noll M. (2017). Using a structural root system model to evaluate and improve the accuracy of root image analysis pipelines. *Frontiers in Plant Science*.

[45] Lazar M. D., Worrall W. D., Peterson G. L. (2004). Registration of ‘TAM 111’ Wheat. *Crop Science*.

[46] R Core Team (2018). *R: A Language and Environment for Statistical Computing*.

[47] RStudio R. T. (2016). *Integrated Development for R*.

[48] Wickham H. (2007). Reshaping data with the reshape package. *Journal of Statistical Software*.

[49] Wickham H. (2016). *ggplot2: Elegant Graphics for Data Analysis*.

[50] Falconer D., Mackay T. (1996). *Introduction to Quantitative Genetics*.

[51] Bates D., Mächler M., Bolker B., Walker S. (2015). Fitting linear mixed-effects models Usinglme4. *Journal of Statistical Software*.

[52] Bucksch A., Atta-Boateng A., Azihou A. F. (2017). Morphological plant modeling: unleashing geometric and topological potential within the plant sciences. *Frontiers in Plant Science*.

[53] Li M., Frank M. H., Coneva V., Mio W., Chitwood D. H., Topp C. N. (2018). The persistent homology mathematical framework provides enhanced genotype-to-phenotype associations for plant morphology. *Plant Physiology*.

[54] Lee U., Chang S., Putra G. A., Kim H., Kim D. H. (2018). An automated, high-throughput plant phenotyping system using machine learning-based plant segmentation and image analysis. *PLoS One*.

[55] York L. M. (2019). Functional phenomics: an emerging field integrating high-throughput phenotyping, physiology, and bioinformatics. *Journal of Experimental Botany*.

[56] Venables W. N., Ripley B. D. (2013). *Modern Applied Statistics with S-PLUS*.

