# Supplementary material for: RhizoVision Crown: An Integrated Hardware and Software Platform for Root Crown Phenotyping
Source: Plant Phenomics. 2020 Feb 15;2020:3074916. doi: 10.34133/2020/3074916 (PMC7706346; doi:10.34133/2020/3074916)
Supplement: Supplementary Materials — Supplementary Material 1 is included as a PDF containing images of the completed hardware platform from different angles, schematic drawings, and a part list for the aluminum structure. A table of the means of all extracted features for the four wheat and soybean parents is found in Supplementary Material 2. A table containing the contribution rates, or proportions of variance, from each principal component and the loadings of all features onto the components for both species is found in Supplementary Material 3. [file 3074916.f1.zip › Supp1_RhizoVision_Crown_Hardware_Plans_Details.pdf]

Hardware plans, images, and details (plans and parts last 2 pages)

RhizoVision Crown: An Integrated Hardware and Software Platform for Root Crown Phenotyping

Anand Seethepalli (primary software developer), Haichao Guo, Xiuwei Liu, Marcus Griffiths, Hussien Almtarfi, Zenglu Li, Shuyu Liu, Alina Zare, Felix B. Fritschi, Elison B. Blancaflor, Xue-Feng Ma, Larry M. York (primary hardware developer)

A

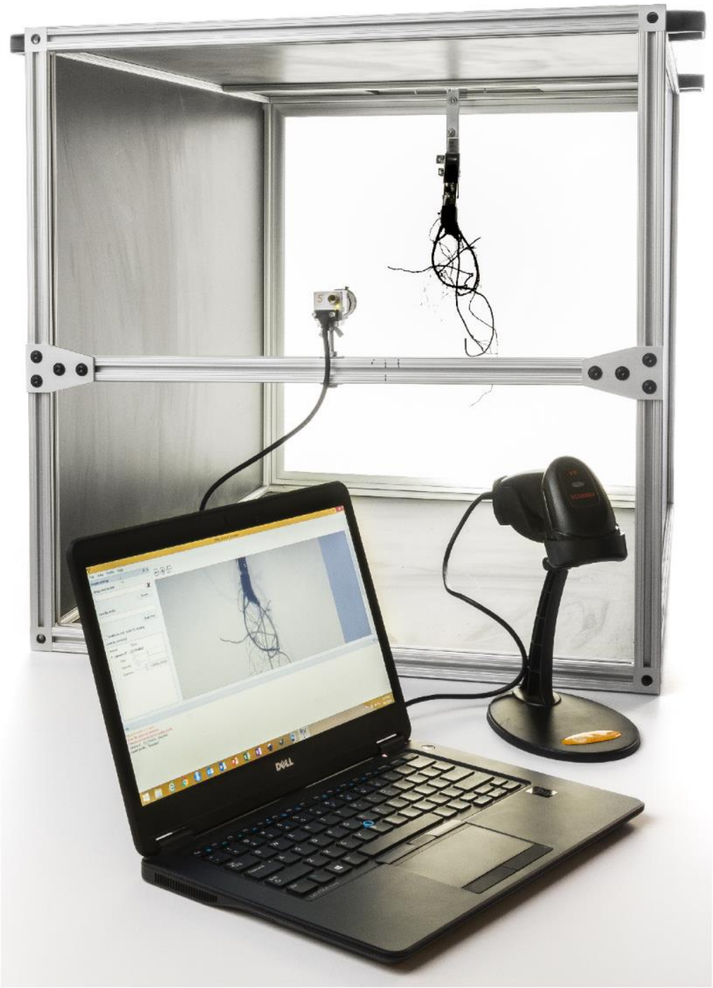

B

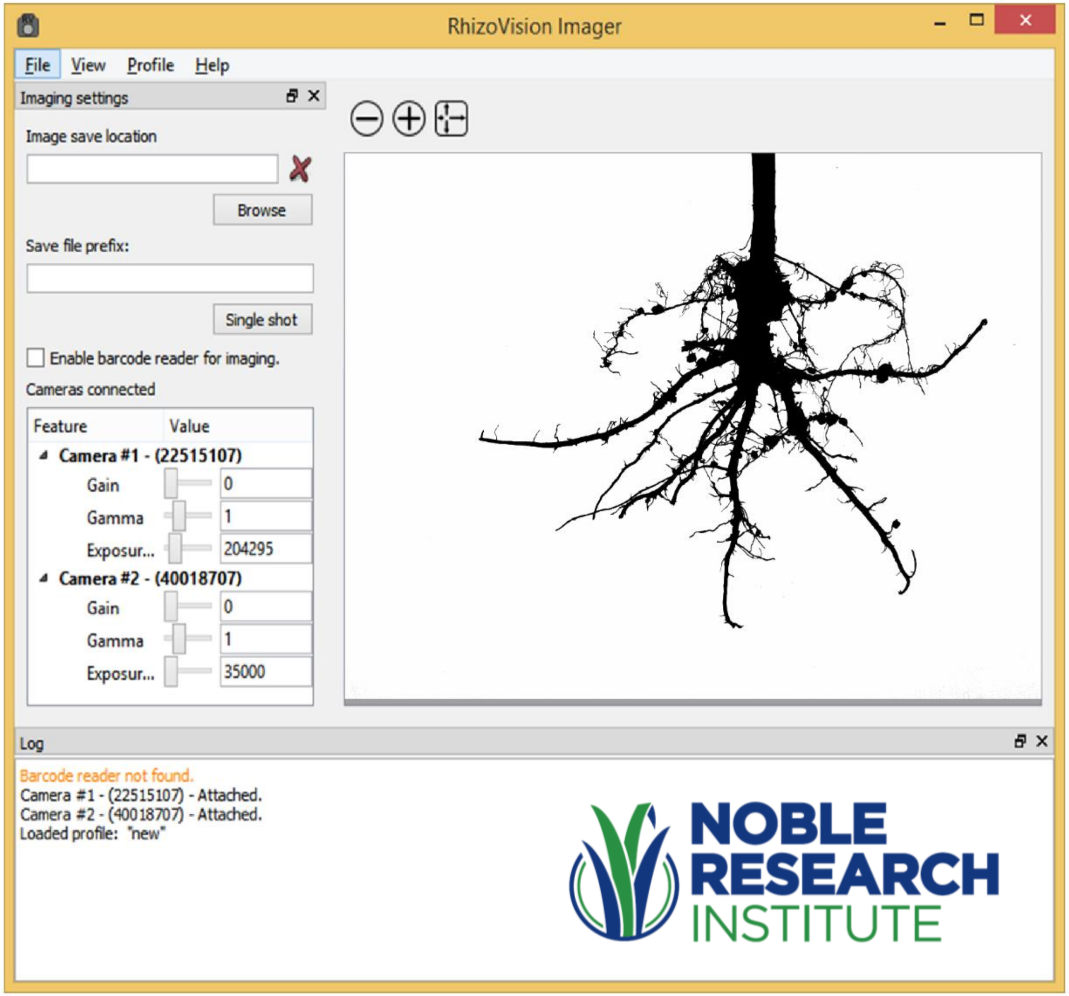

7-hole plates on the back were used to seat light panel then adhesive was added around the interior to keep it in place. Added dimmer but not useful.

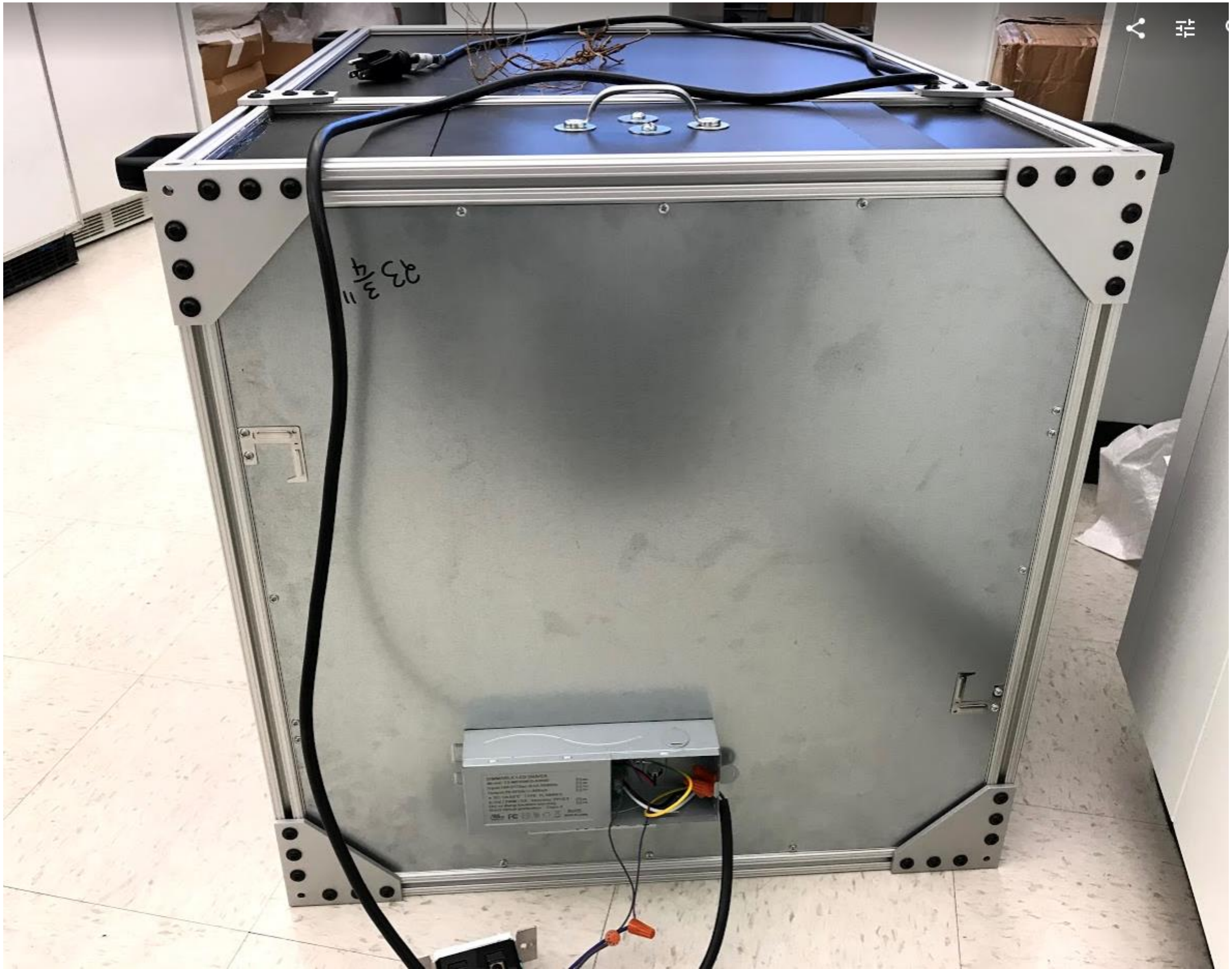

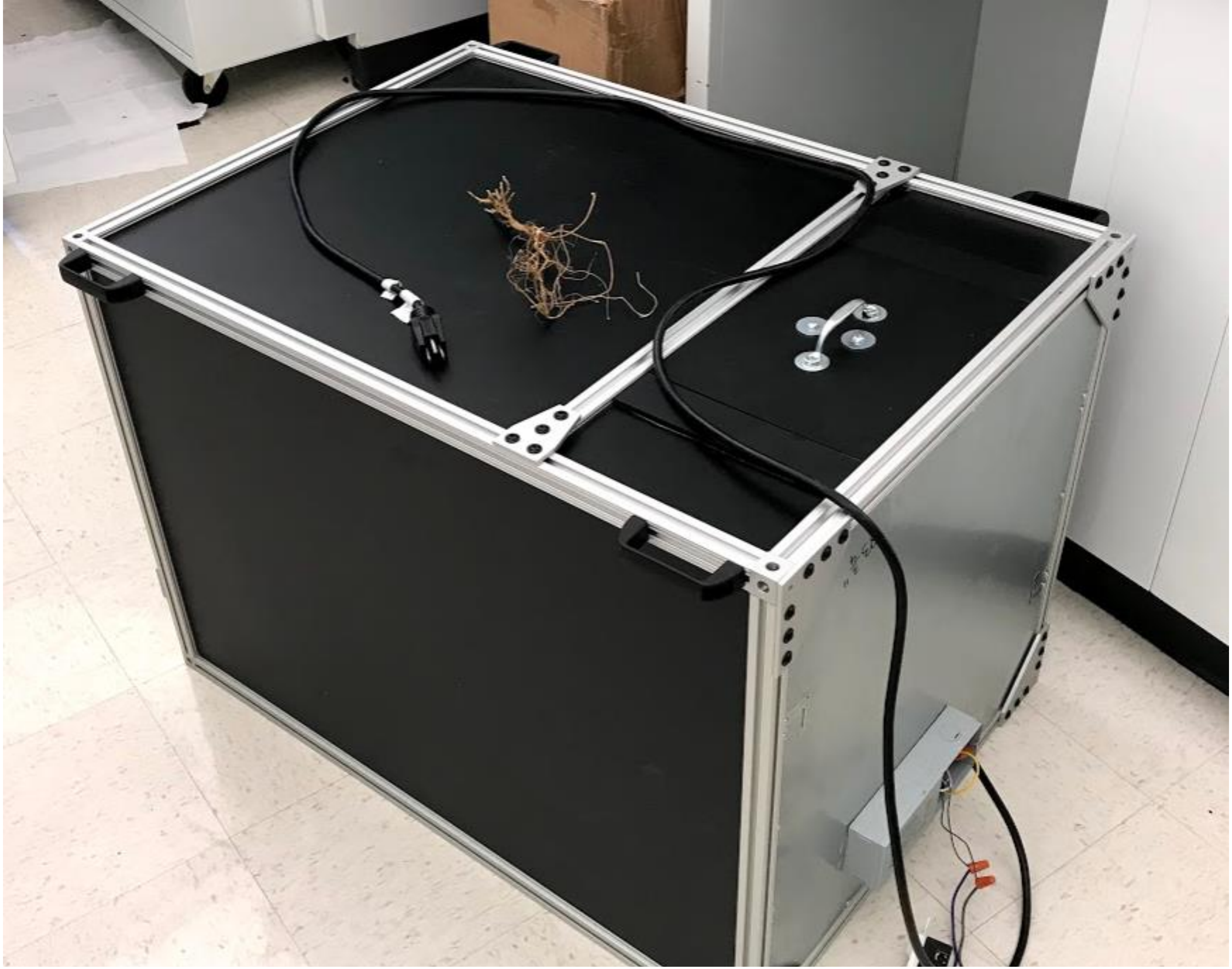

Note this version used corner connectors rather than end-fasteners. The included plans are for use of end fasteners to save money.

Here you can see two ways to mount the camera (ask for the ¼ 20 adapter for the Basler camera to mount on tripod post), either in portrait or landscape.

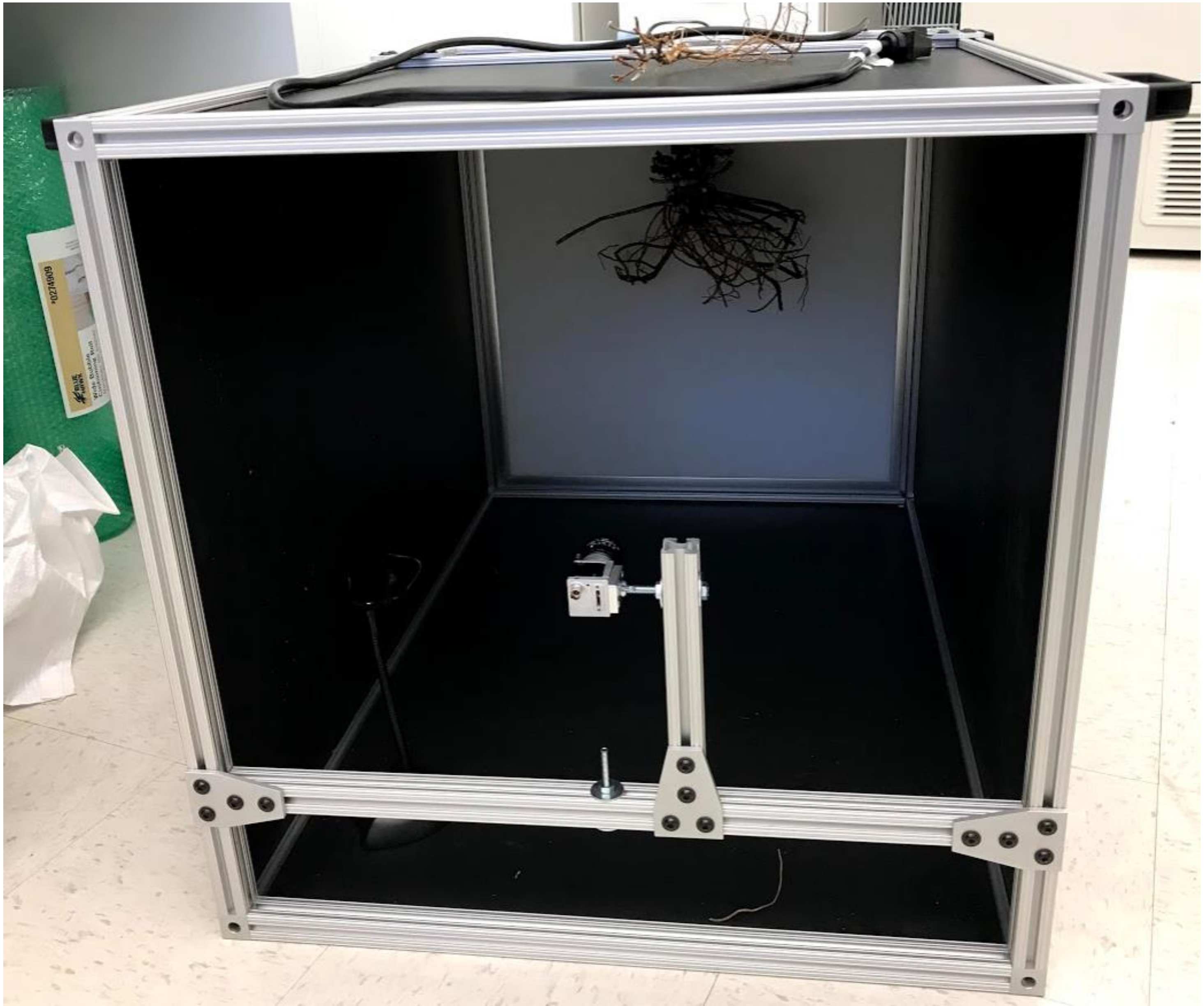

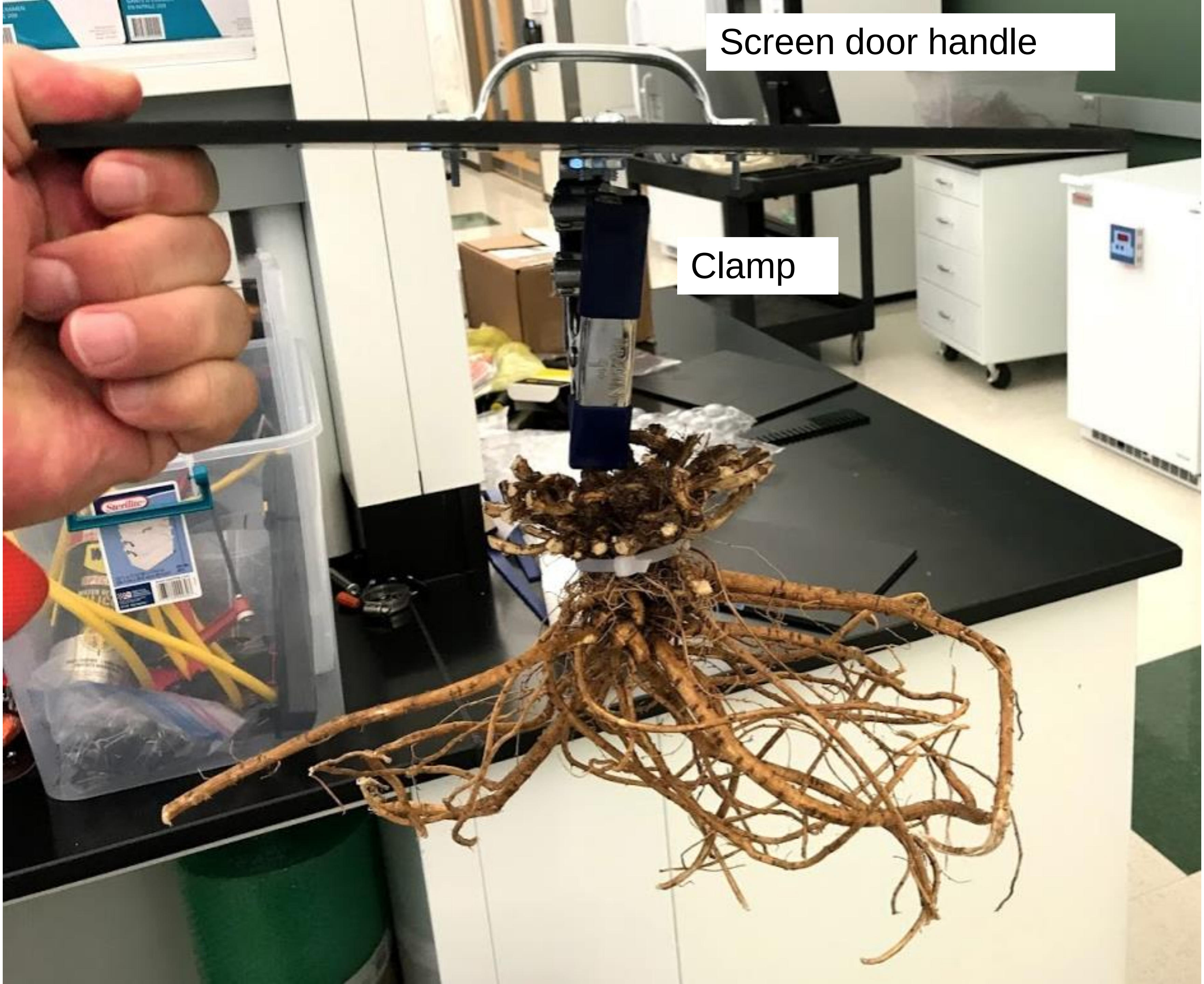
Screen door handle
Clamp

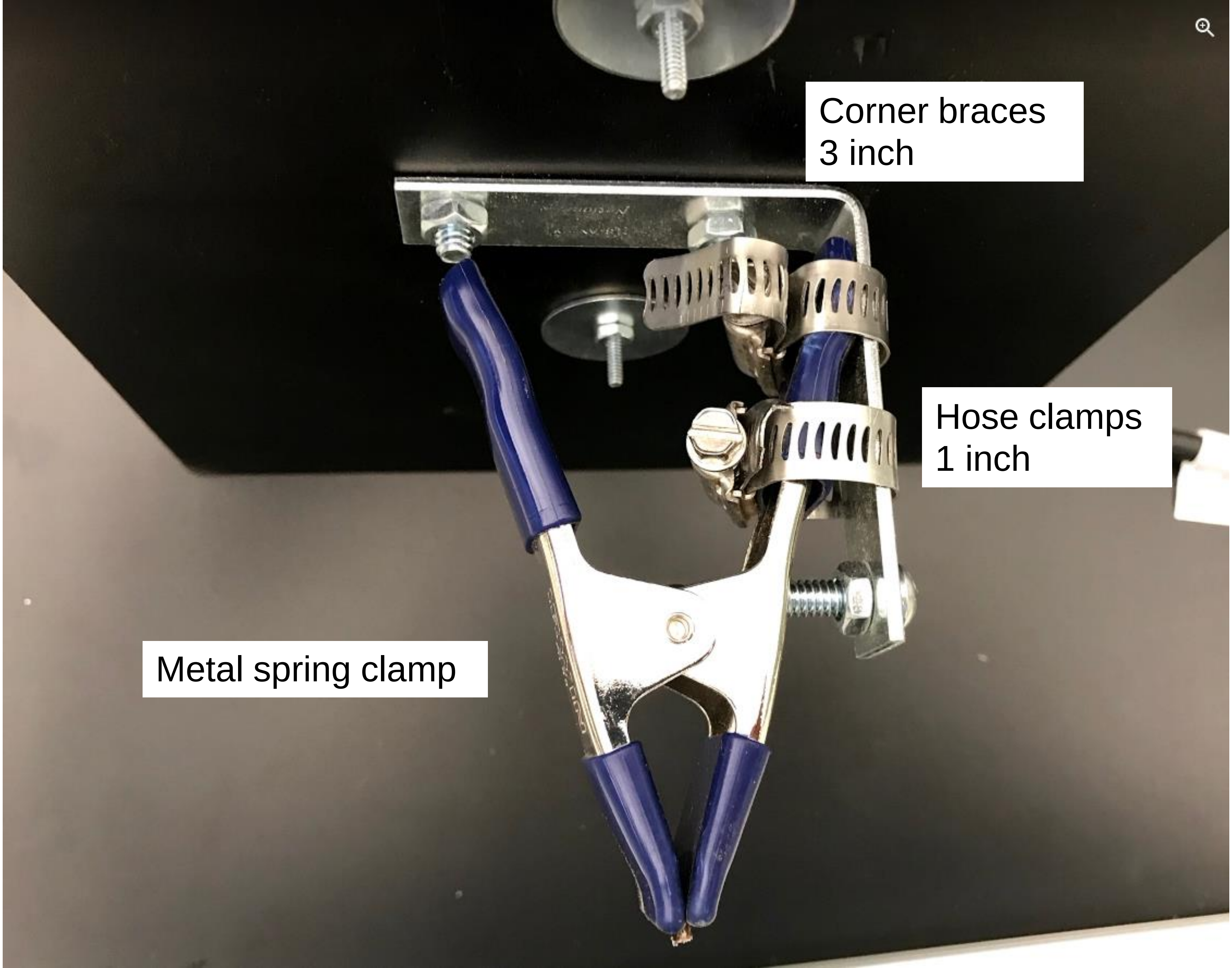
Corner braces
3 inch
Hose clamps
1 inch
Metal spring clamp

These ‘guide’ panels are glued to the side so the roots are always positioned correctly, dimensions give in PDF

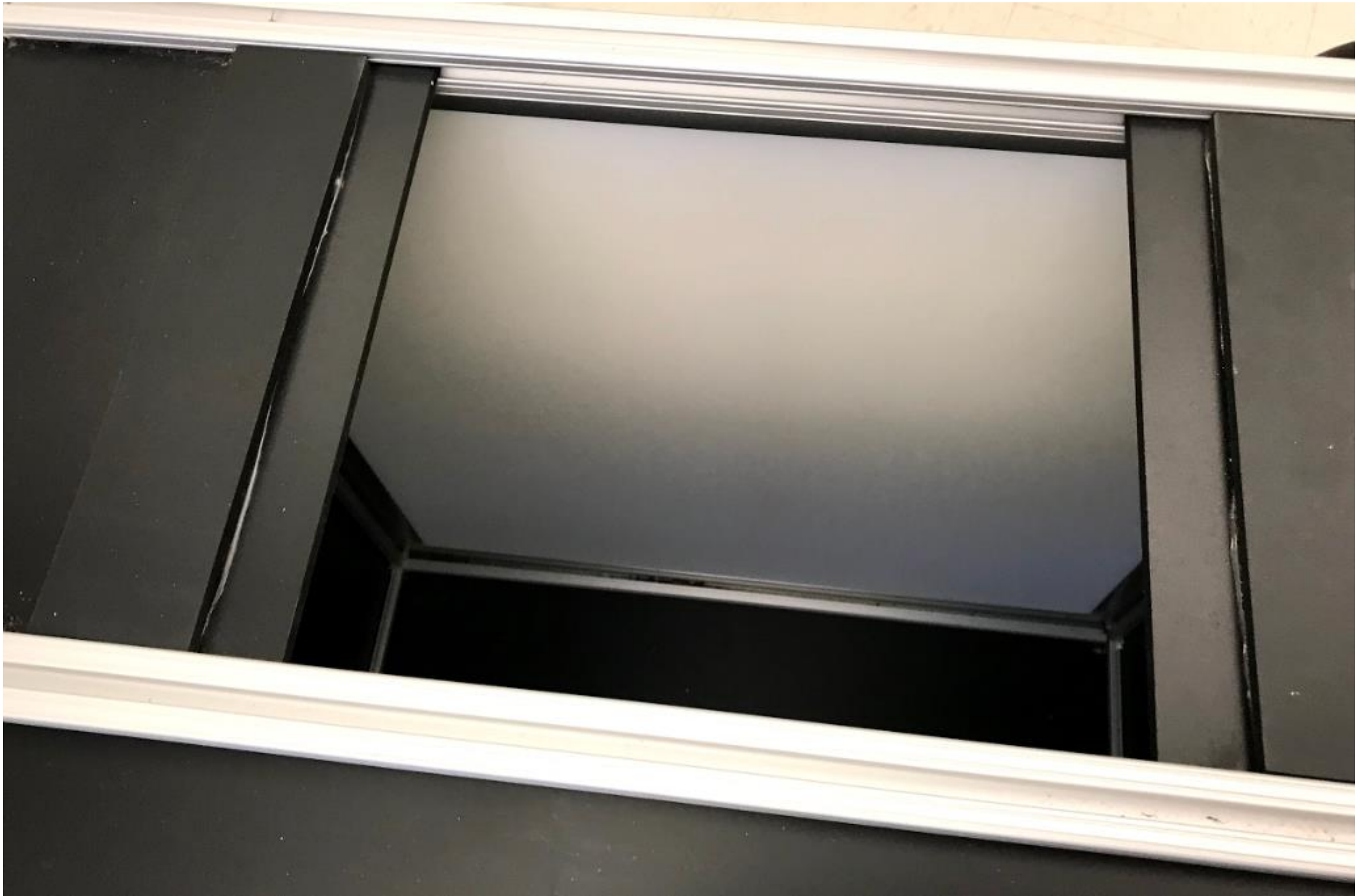

RhizoVision Crown

Revision 4, adjust plastic panel sizes and cross profiles

Larry York, Noble Research Institute

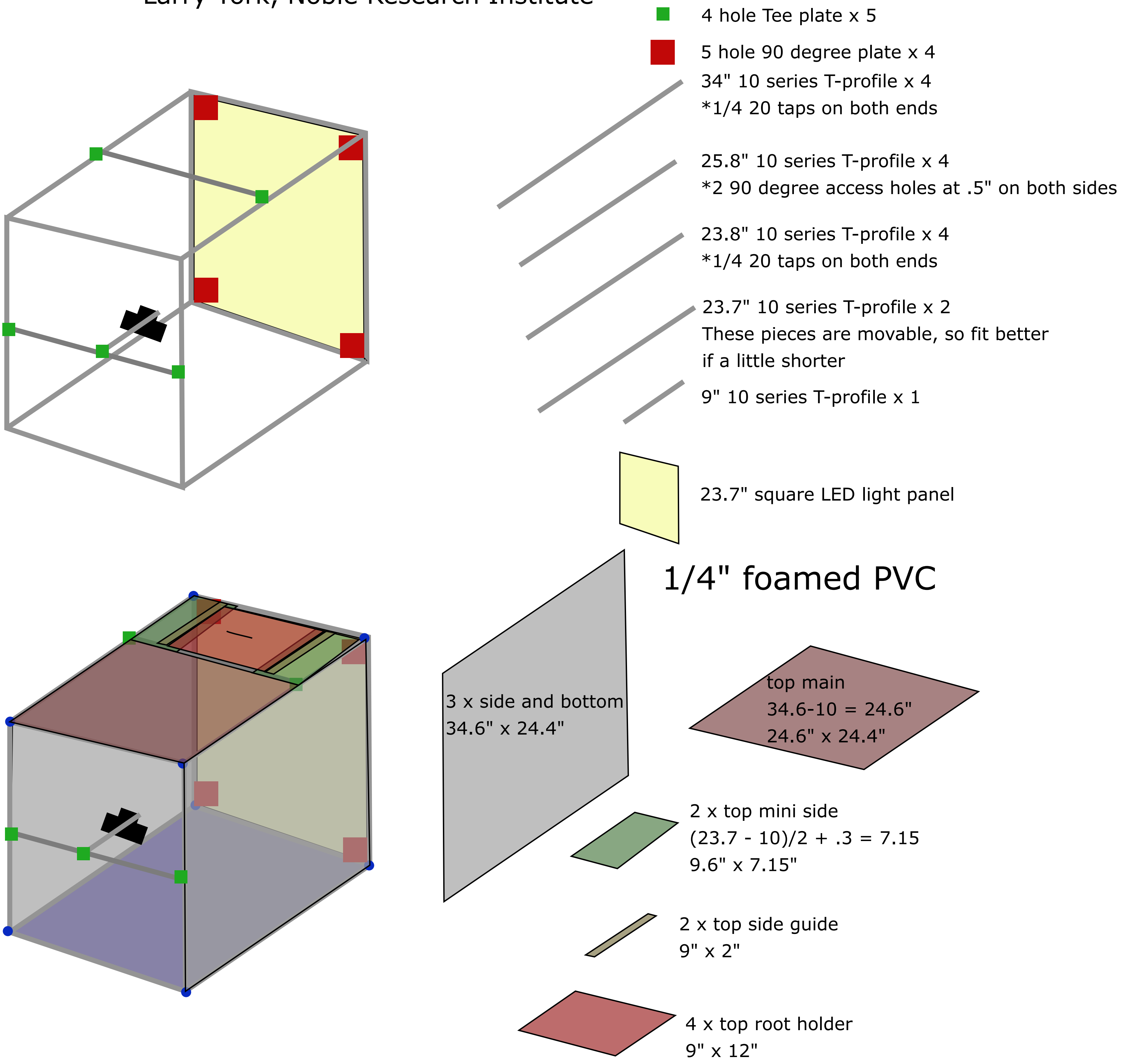

Shopping Cart

Proceed to Checkout →

Continue Shopping

Please visit the Shipping Schedule for more information

Image	Product Name	Machining Services / Each	Price	Qty	Subtotal	Remove
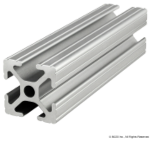 **SKU: 1010** Move to wishlist	**34 Inches (863.6 Millimeters) 1.00" X 1.00" T-Slotted Profile - Four Open T-Slots** **Part Number: 1010**	**1 Cut to Length** **2 End Taps** 7061 Left 7061 Right	**$13.67**	4 update	**$54.68**	Remove Item
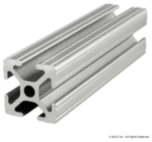 **SKU: 1010** Move to wishlist	**25.9 Inches (657.86 Millimeters) 1.00" X 1.00" T-Slotted Profile - Four Open T-Slots** **Part Number: 1010**	**1 Cut to Length** **4 Access Holes** 7051 in R @ 0.5 7051 in R @ 25.4 7051 in S @ 0.5 7051 in S @ 25.4	**$15.71**	4 update	**$62.83**	Remove Item
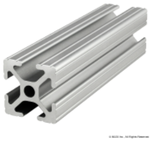 **SKU: 1010** Move to wishlist	**23.9 Inches (607.06 Millimeters) 1.00" X 1.00" T-Slotted Profile - Four Open T-Slots** **Part Number: 1010**	**1 Cut to Length** **2 End Taps** 7061 Left 7061 Right	**$11.35**	4 update	**$45.39**	Remove Item
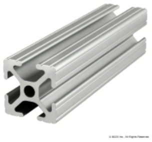 **SKU: 1010** Move to wishlist	**23.9 Inches (607.06 Millimeters) 1.00" X 1.00" T-Slotted Profile - Four Open T-Slots** **Part Number: 1010**	**1 Cut to Length**	**$7.45**	2 update	**$14.89**	Remove Item
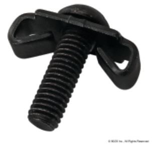 **SKU: 3381** Move to wishlist	**Standard End Fastener, 1/4-20** **Part Number: 3381**	None	**$1.50**	16 update	**$24.00**	Remove Item
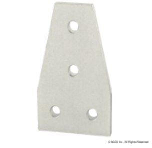 **SKU: 4141** Move to wishlist	**10 Series 4 Hole - Tee Flat Plate** **Part Number: 4141**	None	**$5.80**	4 update	**$23.20**	Remove Item
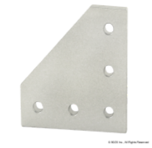 **SKU: 4151** Move to wishlist	**10 Series 5 Hole - 90 Degree Angled Flat Plate** **Part Number: 4151**	None	**$6.30**	4 update	**$25.20**	Remove Item
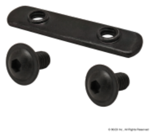 **SKU: 3356** Move to wishlist	**Bolt Assembly: (2) 1/4-20 x .500" Black FBHSCS and Double Slide-In Economy T-Nut - Black Zinc** **Part Number: 3356**	None	**$0.95**	16 update	**$15.20**	Remove Item

Update Shopping Cart

Subtotal: $265.39

Grand Total $265.39

Proceed to Checkout →

Code Apply Coupon

Please visit the Shipping Schedule for more information
